# Books

**Published:** 1881-11

**Authors:** 


### Books.

A System of Surgery, in Treatises by Various Authors. Edited by T. Holmes, M. A. Cantab. First American, from Second English Edition. By Jno. H. Packard, A.M., M.D., assisted by a large corps of American Surgeons. Philadelphia: Henry C. Lea's Son & Co. 1881.

This is the first of three volumes to be issued, based on the English work recently brought out. The English edition embraced six volumes, though smaller than the American issue. The American profession is familiar with the English work of ten or twelve years since.

Among the authors we find such names as Sir James Paget, F.R.S., Henry Arnott, Holmes Cootes, Esq., T. Holmes, Esq., Geo. W. Callender, Esq , A. W. Barclay, Prescott Hewett, Esq., and several others. The editing of the several articles has also been done by different writers in the American edition. We find here the work of such men as J. Henry, C. Simes, Wm. Hunt, J. B. Roberts, Jas. Nevins Hyde, Morris Longstreth, P. S. Conner, T. G. Morton, Sam'l Ashhurst, L. A. Stimson, Jno. H. Packard, J. S. Jewell, Roberts Bartholow, Jno. A. Lidell, Chas. T. Hunter, Jno. T. Hodgen, E. T. Carswell. With the exception of two chapters, the text of the English edition has been reproduced. The additions by the American editors are inserted in brackets.

This volume is devoted to inflammation, traumatic fever, collapse, scrofula, syphilis, tumors and cancer, injuries in general and their complications, and regional injuries.

The arrangement, in some respects, is peculiar. Special fractures or dislocations, for instance, are treated of, not under one head, but as regional injuries. While some advantage may be claimed for this arrangement, we cannot regard this change as an improvement over the more usual order.

All the subjects are elaborately handled. As before said, the American notes are in brackets. It is not pleasant to study a subject with the consciousness throughout that, at the close, the views may be all upset or modified by foot notes. It would be not easy to remedy the objection, but it nevertheless is one.

While treating of inflammation, Lister's method is only briefly alluded to, but with the intimation that the third volume will supply the deficiency.

The chapter on syphilis, while excellent, is not so clearly given as is desirable at this day. The English and Continental views do not really seem so clearly cut on this subject as they are in America; and hence some confusion. Treatment by sufficient doses of mercury and iodide of potassium is very properly urged.

On page 440 we find that a few well authenticated cases of recovery from hydrophobia are conceded. On page 570 we find that there is "no authentic case of recovery."

In collapse from severe shock, it is recommended to open the veins of the neck, provided there be no hemorrhage, the veins are full and the heart has stopped its beat. If the heart still beats, artificial respiration must be resorted to. We believe this is good advice, though the principles on which it depends are often overlooked.

In the treatment of tetanus, chloral is exalted above all other agents. It is to be given early and freely. "As much as 1,120 grains have been given in twenty-four hours." Many other remedies are spoken of but not strongly endorsed.

For lack of space we cannot refer to the various chapters as they deserve. All the subjects are elaborately given. If books must continue to grow in number and size as they have for the past few years, we must extend our sympathy to the students and doctors of the future, in the labor they must put forth to master them. We find we have been disposed to point out the deficiencies of the work; but these are really few, and not glaring. It is a fine work for reference, that covers the ground thoroughly, and one which any physician may rely upon confidently.

The book is very well printed, in double column; the illustrations are fair, and the binding elegant.

---

THE APPLIED ANATOMY OF THE NERVOUS SYSTEM. By Ambrose L. Ranney, A.M., M.D. New York: D. Appleton & Co. 1881. Received through S. P. Richards, Atlanta.

This is a work of five hundred pages, in the form of lectures delivered before the class of the University of New York. It is written in an easy and attractive style. The physiology of the nerves are not discusssd, further than their function as applied in their anatomical distribution, or in points applicable to clinical studies. A number of "axioms" are formulized, as guides in diagnosing disordered nerve action.

The grouping of muscles according to nerve-distribution is elaborately done, and in an interesting manner. The effect of paralysis of a given nerve or nerves is well presented. The "clinical points" are also valuable.

The first half of the work is devoted to the cranial nerves; the latter to those of the spinal cord. In connection with the first, we find indications, fairly given, for trephining the skull for brain lesion.

The illustrations are very fine. The work is well indexed. The printing is well done, and the book attractively bound. There is but little in the work really new; but facts already known are presented in a useful way. The book, on the whole, is a valuable one.

---

GENERAL MEDICAL CHEMISTRY FOR THE USE OF PRACTITIONERS OF MEDICINE. By R. A. Witthaus, A.M., M.D., New York: Wm. Wood & Co. 1881. Wood's Library of Standard Medical Authors.

The author has disarmed criticism by frankly stating that he has seen fit to depart at certain points from the methods usually followed in chemical text books. "Those portions treating of technical processes have been condensed to a minimum, while the bearings of chemistry upon physiology, hygiene, therapeutics and toxicology have been treated of as fully as the limits of the book have permitted."

His classification is somewhat obscure, and in some particulars fanciful, not to say arbitrary.

The absence of technical detail, illustrations, etc., will prevent its adoption as a text book for students who attend medical lectures, who must learn the rudiments of didactic and anlytical chemistry. But issued as it is in the midst of a series of books on the subscription plan, it was no doubt prepared for the class its title indicates, medical practitioners, who are supposed to be already possessed of some knowledge of the subject, and only desire to obtain the latest developments of the science as applied to the problems of medicine, and recall in a general way information they already possess. To such it will no doubt prove valuable.

Barring a few unimportant typographical errors, the printing and binding is all that could be desired, and sustains the well earned reputation of the publishers.

W. D. B.

---

TRANSACTIONS OF THE AMERICAN GYNECOLOGICAL SOCIETY. Volume V. For the year 1880. Boston: Houghton, Mifflin & Co. 1881.

Besides a list of officers, fellows, honorary fellows and the proceedings of the fifth annual meeting of the society, this volume contains the address of the president, Dr. J. Marion Sims, and a number of interesting and valuable papers.

Dr. Robert Battey contributes a paper on "What is the proper field for Battey's operation?" Dr. Joseph A. Eve a paper on "Occlusion of the gravid uterus" and Dr. Henry F. Campbell treats of "Quinine in gynecic and obstetric practice." Among the other contributions is a profusely illustrated paper on "Posture in labor," by Dr. George J. Engleman. "Secondary puerperal metrorrhagia," by Dr. Theophilus Parvin. "Successful extirpation of an encephaloid kidney," by Dr. William H. Byford. "Uterine massage as a means of treating certain forms of enlargement of the womb," by Dr. A. Reeves Jackson. "Ovariotomy during pregnancy," by Dr. Henry P. C. Wilson, and "Manual dilatation of the os uteri as a means of inducing premature labor," by Dr. William L. Richardson.

The book is made useful for reference by the presence of a general index to obstetric and gynecological literature of all countries for 1879, prepared with the co-operation of Dr. J. S. Billings; an index of obstetric and gynecological journals; and an index of obstetric and gynecological societies.

---

INDEX CATALOGUE OF THE LIBRARY OF THE SURGEON GENERAL'S OFFICE. United States Army. Authors and subjects. Volume II. Berlioz.—Cholas. Washington: Government Printing Office. 1881.

This volume represents an enormous amount of painstaking labor. Some idea of the magnitude of the work may be formed when we state that this single volume, of over one thousand pages, only contains alphabetical reference to names and titles from "Berlioz" to "Cholas." The complete work will consist of ten volumes of same size and arrangement. The number of copies available for distribution will be small, but under existing laws, any one can obtain, from the government printer, copies of the work at cost, plus ten per cent. The estimated cost is for paper, press work and binding in cloth. It amounts to about $2.00 per volume.

